# Prevalence of iron deficiency in a South African adolescent inpatient psychiatric population: Rates, risk factors and recommendations

**DOI:** 10.4102/sajpsychiatry.v25i0.1347

**Published:** 2019-07-16

**Authors:** Theonie du Plessis, Karis Moxley, Anusha Lachman

**Affiliations:** 1 Department of Psychiatry, Faculty of Medicine and Health Sciences, Stellenbosch University, Cape Town, South Africa

**Keywords:** Iron deficiency, Anaemia, Psychiatric illness, Adolescent, Risk factors

## Abstract

**Background:**

Severe iron deficiency is associated with anaemia, but iron deficiency with normal haemoglobin (Hb) may also affect morbidity and quality of life and contribute to psychiatric illness onset and severity. Psychiatric presentations in adolescence are often indicative of serious long-term morbidity, and addressing contributing health risk factors, such as iron deficiency, is important.

**Objectives:**

To determine rates of iron deficiency in a South African inpatient adolescent psychiatric population and possible associations between psychiatric diagnosis and iron deficiency risk factors.

**Methods:**

We conducted a retrospective chart review of all adolescent patients (13–18 years old) who were admitted to the Adolescent Psychiatric Inpatient Unit at Tygerburg Hospital (Cape Town, South Africa) during 2016. Patient records were limited to those with haemoglobin and ferritin levels available, as well as a psychiatric disorder diagnosed according to the Diagnostic and Statistical Manual of Mental Disorders. The final sample consisted of 93 patients.

**Results:**

Of all participants, 7.6% were anaemic, while 22.6% were iron deficient. We found 29% of our population to have anaemia in the absence of iron deficiency. Gender was the only statistically significant correlate, with adolescent females at particular risk of compromised iron status as indicated by a low ferritin level (45% of female sample).

**Conclusion:**

Iron deficiency rates remain a relevant health concern, and testing Hb alone is inadequate to assess iron status in this population. Ferritin is a necessary additional parameter and should be included in the usual medical workup.

## Introduction

Psychiatric illness often becomes symptomatic during adolescence and early adulthood. Adolescent onset of psychiatric illness is associated with more severe illness, higher rates of relapse and worse long-term outcomes.^1^ Therefore, early identification of possible contributing or predisposing health risk factors is relevant for this highly vulnerable population group. In particular, adolescence has been identified as a time of particular physiological vulnerability for deficient iron stores, especially in females.^2^ Iron deficiency and iron deficiency anaemia have been associated with an increased risk and earlier onset of childhood and adolescent psychiatric illness, including unipolar depression,^3^ bipolar disorder,^4^ anxiety disorders,^5^ autism spectrum disorders,^6^ attention deficit hyperactivity disorders,^7^ neurodevelopmental delays and learning disabilities.^8^

Although the precise relationship between iron deficiency and the development of psychiatric disorders is poorly understood, iron is known to be an essential element for normal neurological function and is essential for brain development.^9^ Iron deficiency affects the availability of dopamine, noradrenaline and serotonin in various areas of the brain^10^ and has been associated with delayed psychomotor development in infants and impaired cognitive performance throughout childhood and adolescence.^11^ Iron deficiency can contribute to general morbidity in child and adolescent health,^12^ and it contributes to poor sleep quality, lethargy and potential long-term behavioural changes.^13^ Adolescents are recognised as a nutritionally ‘at-risk’ group, as they have high nutritional demand for growth, poor eating behaviour as well as a propensity for unhealthy behaviours. Substance use in adolescence is considered an escalating health risk, and this may be a contributor to the nutritional challenges in this population. A study by Naude reported iron store depletion in the form of serum ferritin in a quarter of adolescents both with and without alcohol use disorders, indicating a greater risk of iron store depletion in this population.^14^

Iron deficiency is recognised as one of the most common and relevant nutritional deficiencies worldwide. The World Health Organization (WHO) highlights iron deficiency as an important public health issue, and global prevalence of anaemia is estimated to be more than 30%.^15^ The prevalence of iron deficiency and iron deficiency anaemia is estimated to be 2% – 6% among children in Europe^13^; however, low- to middle-income (LMIC) countries are at higher risk because of poor nutrition and health resource service limitations.^15^ Up to 50% of pregnant women and 40% of preschool children in LMICs are estimated to be iron deficient or anaemic.^1^ According to a recent South African National Health and Nutrition Examination Survey Report,^16^ the rates of iron-deficiency anaemia in the country have improved when compared to data from previous national surveys, specifically for children under 5 years old. However, there are limited data available concerning the prevalence of iron deficiency among adolescents, both locally and abroad.

Considering the morbidity and quality of life issues associated with adolescent onset of psychiatric illness, as well as the high prevalence of childhood malnutrition in South Africa,^17^ the diagnosis and prevention of iron-deficiency and iron-deficiency anaemia are vital for adolescent health. Despite this, there is currently no routine screening procedure for iron levels among adolescents. Standard practice at tertiary South African facilities is to request iron tests only for those individuals considered by the treating physician to be ‘at-risk’. Furthermore, when iron tests are indicated, there is little consistency as to which components of the iron studies are requested.

Although there is an array of different tests for haemoglobin (Hb), there is no ‘best’ approach for iron screening. Bone marrow aspiration is the ‘gold standard’ for the diagnosis of iron deficiency, but it is too invasive for routine use.^18^ Therefore, indirect assays are preferred. In the absence of inflammation, serum ferritin is widely agreed to be the most specific non-invasive biochemical test to diagnose iron deficiency and is an accurate reflection of total body iron stores.^19^ Assaying ferritin levels is useful because a reduction in this marker occurs before detectable reductions in Hb, serum iron levels or erythrocyte size, which traditionally indicate the presence of anaemia at more severe stages of iron deficiency. Furthermore, when compared to bone marrow aspiration, the sensitivity of using ferritin to assess iron deficiency is 98% for a cut-off value of 30 µg/L.^20^ Based on this, screening for iron deficiency among adolescents via ferritin testing may be suitable for routine implementation in South African healthcare settings.

However, one of the many challenges in a resource-constrained environment is the provision of evidence-based healthcare (including relevant laboratory investigations) that is appropriate and clinically cost-effective. Routine screening for only ferritin and haemoglobin would certainly be more cost-effective than the complete iron studies that are often requested, but the implementation of such a strategy depends on the provision of suitable motivating evidence. That is, in order to motivate for the inclusion of mandatory ferritin and haemoglobin screening upon admission, there is first a need to quantify the prevalence of iron deficiency among South African adolescents. Therefore, the aim of this study was to determine the iron status of adolescents presenting for treatment at the Adolescent Inpatient Psychiatry Unit at Tygerburg Hospital. In addition, possible associations with demographic and clinical risk factors for iron deficiency and psychiatric illnesses were explored to determine any additional factors that may contribute to iron deficiency in the local setting.

## Methods

### Study design

In 2017, we performed a retrospective chart review of patients referred to the Adolescent Psychiatric Inpatient Unit at Tygerburg Hospital (TBH; Cape Town, South Africa) between 01 January 2016 and 31 December 2016.

### Study setting

Tygerberg Hospital is a tertiary level hospital situated in a peri-urban area in Cape Town, South Africa. The Adolescent Psychiatry Unit receives referrals from primary, secondary and tertiary level hospitals and serves a heterogeneous population of adolescents between the ages of 13–18 years. Adolescents are screened by a multidisciplinary team (MDT) that includes a psychiatrist, psychiatry resident doctor, psychologist and psychology intern, occupational therapist, social worker and advanced psychiatry nurse. During the study period, serum ferritin and/or iron levels were routinely requested for patients being treated for psychiatric illness. Standard clinical practice during admission included documentation of clinical history and demographic data as well as psychiatric diagnosis according to the Diagnostic and Statistical Manual of Mental Disorders (DSM).^21^

### Sample

A total of 151 adolescents (13–18 years old) were seen at the inpatient unit during the study period. For the purposes of this study, records were limited to 126 patients who had haemoglobin (Hb) and ferritin levels available in their charts, as well as a psychiatric disorder diagnosed according to the DSM-5.^21^ We also excluded patients who were pregnant or postpartum, as well as patients diagnosed with an eating disorder.

Furthermore, as ferritin is an acute-phase protein and can be elevated in patients with chronic inflammation or infection, we excluded patients with known chronic illnesses, with infectious diseases (including HIV, hepatitis, TB) or with a C-reactive protein (CRP) level of more than 10. The final sample consisted of 93 patients.

### Data collection

The following data were extracted from the patient files by the principal investigator and subsequently entered into an anonymised Microsoft Excel spreadsheet: gender, age, body mass index (BMI, a standardised measure using height and weight for age), eating patterns, nutritional status, iron supplementation, menstruation history, history of blood loss or helminthic infections, presence of extrapyramidal side effects, substance use disorders and psychiatric diagnoses. Hb values of < 12 g/dL in adolescent females and < 12.5 g/dL in adolescent males were used to diagnose anaemia.^22^ We used a cut-off ferritin level < 30 µg/L to indicate iron deficiency. When multiple blood work data were available, only data from the first blood sample taken during the study period were used for research purposes. Demographic and clinical information reflect the patients at the time this blood sample was taken.

### Statistical analysis

Demographic and clinical data were summarised as means with standard deviation for continuous variables and as counts with percentages for categorical variables. Independent *t*-tests or Fisher’s exact test were used to test for differences in demographic and clinical variables between males and females. Quantile regression (median regression) was used to assess the possible relationship between Hb or ferritin levels with demographic and clinical variables. Risk differences and 95% confidence intervals were estimated. All analyses were performed using SPSS, version 25 (IBM Corp., Armonk, N.Y., USA), and statistically significant differences were established at *p* < 0.05.

## Ethical consideration

The study was approved by the Health Research Ethics Committee of Stellenbosch University (study number: S16/07/139), and the authors were granted a waiver of informed consent. Additional site approval was granted by Tygerberg Hospital. All data were anonymised using a study number to code for each folder number. All data were stored in a Microsoft Excel spreadsheet and kept on a password-protected computer in a secured area.

## Results

Of all patients (*N* = 93), 41% were female and 59% were male (Table 1). The mean ± s.d. age at presentation was 15.84 ± 1.34 years. Females (mean age ± s.d., 15.5± 1.5 years) were significantly younger (Student’s *t*-test; *p* = 0.024) than males (mean age ± s.d., 16.1 ± 1.2 years). More than half the sample (58.1) smoked cigarettes, and far more males (40.9%) smoked compared to females (17.2%). Table 1 shows the prevalence of different psychiatric diagnoses and their distribution between males and females. Cannabis use disorder was the most prevalent (53.7%) psychiatric diagnosis. Schizophrenia and schizophreniform disorders were the second most prevalent psychiatric diagnoses (33.3%), followed by bipolar disorder and substance use-induced psychotic disorder (both 15.1%). Multiple comorbidities were common in this sample.

**TABLE 1:** Frequency, *n* (%), of psychiatric diagnoses among adolescents (*N* = 93), recorded at discharge.

Diagnosis	Overall (*N* = 93)†		Gender			
			Males (*n* = 55)†		Females (*n* = 38)†	
	*n*	%	*n*	%	*n*	%
Cannabis use disorder	50	53.7	37	39.8	13	14.0
Methamphetamine use disorder	14	15.1	11	11.8	3	3.2
Alcohol use disorder	10	10.8	7	7.5	3	3.2
Mandrax use disorder	8	8.6	7	7.5	1	1.1
Schizophrenia and schizophreniform disorder	31	33.3	24	25.8	7	7.5
Bipolar disorder	14	15.1	9	9.7	5	5.4
Substance-induced psychotic disorder	14	15.1	10	10.8	4	4.3
Neurodevelopmental disorders‡	11	1.8	8	8.6	3	3.2
Major depressive disorder	10	10.8	4	4.3	6	6.5
Post-traumatic stress disorder	9	9.7	0	0.0	9	9.7
Somatic symptom related disorders	5	5.4	0	0.0	5	5.4
Anxiety disorders	5	5.4	3	3.2	2	2.2
Obsessive compulsive disorder and related disorders§	2	2.2	0	0.0	2	2.2
Conduct disorder	3	3.2	2	2.2	1	1.1
Substance-induced mood disorder	1	1.1	1	1.1	0	0.0

† , All percentages are relative to the total sample number (*N* = 93).

‡ , Including intellectual disability and autism spectrum disorder.

§ , Including body dysmorphic disorder.

The average duration of stay in the unit was 35.6 days, and females stayed a week longer than males. Half (47%) of the patients were transferred for on-going inpatient treatment at a step-down inpatient facility after discharge. Fourteen patients (15%) had documented pronounced extrapyramidal side effects (EPSEs), which necessitated treatment with anticholinergic medication and a switch to an alternative antipsychotic medication.

The mean BMI ± s.d. of the sample was 21.2 ± 4.8, and there was no significant difference in BMI between gender groups (Student’s *t*-test; *p* = 0.765). Two patients were on iron supplementation on admission. None of the patients were vegetarians. None of the patients had a history of significant blood loss in the year preceding admission, and there were no blood donors included in the sample. The recorded data relevant to menstruation history were incomplete in the files and were not used as a study variable. None of the patients had a documented history of parasitic worms or recent treatment for parasitic infestations.

The overall prevalence of iron deficiency was 22.6%, and the prevalence among males (7.3%; 95% CI: 2.0 – 17.6%) was much lower than among females (44.7%; 95% CI: 28.6 – 61.7%). We found 29% of our population to have anaemia in the absence of iron deficiency. The overall prevalence of iron-deficiency anaemia was 7.6%. More females (16.2%, 95% CI: 6.2 – 32.0%) were anaemic compared to males (1.8%; 95% CI: 0.1 – 9.7%). Mean ferritin levels were significantly lower (Student’s *t*-test; *p* < 0.001) among females (mean ferritin ± s.d., 50.1 ± 33.1 µg/L) compared to males (mean ferritin ± s.d., 78.1 ± 42.2 µg/L). Mean Hb levels were also significantly lower in females (mean Hb ± s.d., 12.7 ± 0.9 dL) compared to males (mean Hb ± s.d., 14.6 ± 1.2 dL). Quantile regression revealed that, of all demographic and clinical variables tested, only gender was significantly associated with Hb or ferritin levels. In particular, being female was significantly associated with reduced Hb (*p* < 0.001) and ferritin (*p* = 0.01).

## Discussion

We evaluated the prevalence of iron deficiency in a sample of adolescent patients admitted to a tertiary South African adolescent psychiatric inpatient unit. To our knowledge, this is one of the first studies to assess the local prevalence of iron deficiency in a psychiatric sample of adolescents. We found the prevalence of iron deficiency to be 22.6%. This is much higher than the 4.7% prevalence reported for European adolescents,^23^ but similar to a prevalence of 19% reported in other African countries.^17,24^

The prevalence of iron deficiency anaemia in our sample was 7.6%, which is higher than 4.4% typically seen in European studies.^23^ This is likely because of the high prevalence of childhood malnutrition in South Africa.^25^ The South African National Health and Nutrition Examination Survey (SANHANES) found the prevalence rate of anaemia to be 17.5% among adults (over 15 years), with significantly higher rates among females (22%) compared to males (12.2%).^16^ Tygerberg Hospital serves a peri-urban and low- to middle-class population. We presume that better food security among this population might account for the lower rate of anaemia observed in this study.

The SANHANES reported a mean ferritin level of 65.3 µg/L for females,^16^ which is higher than the ferritin level (49 µg/L) we found in this study. This might suggest that the younger female population in our study is at higher risk for iron-deficient states preceding anaemia. The SANHANES study also did not exclude patients with health complications, and this could further explain the raised mean ferritin level compared to that of our group of healthy adolescents as we attempted to exclude patients with medical illness or chronic inflammation.^16^ We found a significant difference in Hb levels between genders, with a mean Hb of 14.6 g/dL for males and 12.7 g/dL for females. These data are very similar findings to the SANHANES, which reported Hb levels of 14.7 g/dL and 12.8 g/dL among males and females, respectively.

It is not clear why 29% of our sample was anaemic in the absence of iron deficiency, because none of the patients were vegetarians or blood donors, and none had a history of significant blood loss or parasitic infestations. Future studies need to take into account full dietary and menstruation history and should also screen for parasitic infections. It is also important to keep in mind that only 45% of South African residents are food secure,^8^ and there is a high prevalence of childhood malnutrition in the country.^25^ These factors are likely to have impacted the eating patterns and nutritional status of our participants, who mostly originated from urban informal areas. SANHANES reported that the highest rates of anaemia were among women from urban informal areas.^16^

A noteworthy finding was the presence of EPSEs in 15% of our sample. Some studies have shown that low serum iron levels may be a risk factor associated with the development of EPSEs – especially akathisia in schizophrenia patients treated with antipsychotics.^26^ Furthermore, iron deficiency has been shown to aggravate the symptoms of restless leg syndrome.^27^ However, we found no significant association between EPSEs and ferritin or Hb levels.

It is imperative to note that the most prevalent psychiatric diagnosis in this study sample was cannabis use disorder (53.7%). This is in keeping with a previously described local Western Cape prevalence rate for comorbid substance use at this unit (54%) where cannabis was also the predominantly used substance.^28^

Substance abuse generally leads to a lack of proper nutrition, either as a result of not eating enough throughout the day or eating foods that are low in necessary nutrients. Cannabis is widely recognised as an appetite stimulant, but in chronic users, this may lead to an increased intake of high caloric but nutritionally poor food. The impact of cannabis use on the physical health and nutritional status of adolescents has not been well documented. In a study by Farrow et al., adolescents who abused cannabis reported eating more snack foods, and fewer fruits and vegetables compared with adolescents who abused other substances.^29^

Similar to our study, while there were no significant associations between the haematologic status (transferrin and haemoglobin) and cannabis use, Farrow et al. found that nutritional disability from substance abuse in adolescents was related to more poor dietary habits and symptomatic deterioration in general health.^29^

The study was limited to one site and the sample size was small, which limits the generalisability of our findings. Furthermore, as with any retrospective study, the validity and accuracy of our findings are subject to the quality and consistency of the information available in patient folders. The lack of menstrual history data in the files is a particular shortcoming. We also could not rule out the possible effects of chronic infection (ferritin could be raised as part of the acute-phase reaction) because CRP levels were not requested for all patients. Nevertheless, we can make a number of important recommendations for clinical practice based on our preliminary findings (Box 1). First, we recommend routine testing of iron status in all adolescent females referred for psychiatric assessment. In addition, as ferritin levels are a sensitive indicator of iron status, these should be requested to complement Hb results. We also recommend that CRP should be requested because ferritin levels could be raised as part of the acute-phase inflammation reaction. Ferritin levels should be tested again after 3 months. We also recommend that psychiatric assessment of adolescents should include a detailed history of dietary intake, menstrual patterns and possible helminthic infestation. Finally, treatment plans should include dietary advice and iron supplementation, as needed.

BOX 1 Summary of clinical recommendations.

**Table:** 

1. Routine testing of iron status in all adolescent females referred for psychiatric assessment is indicated.
2. Ferritin is a sensitive indicator of iron status and should complement the haemoglobin.
3. If infection or inflammation is suspected, include a CRP and be aware that ferritin could be raised as part of the acute-phase reaction. Repeat the ferritin level after 3 months.
4. Psychiatric assessment of adolescents should include a detailed history of dietary intake, menstrual patterns and possible helminthic infestation.
5. Treatment plans should include dietary advice and iron supplementation as needed.

## Conclusion

Adolescence is recognised as a particularly sensitive time in terms of brain development and re-organisation, and iron is known to be essential in many cellular and metabolic processes in the brain. Iron-deficiency, therefore, may have important implications in terms of illness morbidity (including psychiatric morbidity). This reversible nutrient deficiency, as well as iron-deficiency anaemia, was prevalent in our setting, particularly among females. Routine testing of ferritin levels in addition to haemoglobin levels is indicated among psychiatric adolescents. We also recommend that a detailed dietary history should be part of the initial assessment and that dietary recommendations with iron supplementation, where indicated, should form part of comprehensive intervention plans. Overall, this study emphasises the importance of addressing comorbid challenges to optimise health outcomes among psychiatric adolescents.
